# Hospital and Institutional News

**Published:** 1913-03-01

**Authors:** 


### HOSPITAL AND INSTITUTIONAL NEWS.

### THE WEARY WILLIES PROBLEM.

The Hull instance of young and able-bodied men who have become loafers and bullies indicates a problem which presses for practical treatment without delay. Mr. John Burns would do a piece of good practical work if he would bring in a Bill to place the whole of the Weary Willies throughout the country under strict discipline, made to learn some useful trade or occupation, and taught to exhibit self-control and good conduct before being permitted to return to civil life. Weary Willies are like an infectious disease, their presence in the midst of a crowded population multiplies loafers and is only second to the evil caused by the presence of the criminal class. Anyone who has studied the statistics of criminals must realise that punishment and suppression cannot cure the evil. A criminal on leaving prison should be taught a trade, and when he has acquired it, with a full apprehension of his responsibility, he should be found an opening for honest work, and given the opportunity to become a self-contained and respecting man. In the same way the Weary Willies under such a system as this might, for the most part, be redeemed from the decadence and ruin which must otherwise overtake them, whilst the community at large would be spared from grave dangers and evils which at present afflict many innocent people in every great centre of population as well as in some rural districts. The present policy of drift in regard to Weary Willies and to criminals is dishonouring to our day and generation, demoralising to the people at large, and a danger to the community.

### YOUNG AND ABLE-BODIED.

It has been said, with what truth we cannot vouch, that there are over three-quarters of a million of Weary Willies in this country at the present time. If the figures are even approximately correct they point to grave failure in our handling of the problems of education, the training of the youth of the country in habits of self-respect, and providing them with the right kind of knowledge to equip them for the battle of life so that they may readily earn a decent living at an honest trade. That these problems press for solution is proved by the fact that certain young and able-bodied men, nowadays, seem to regard the workhouse as a place which they can always frequent, as and when they choose, which they can enter or leave at their pleasure, and further that they may, by forming themselves into small groups in large towns, enter a workhouse and take charge of the institution. Taking charge means that they band themselves together, and by threats and violence terrorise the labour masters and porters, and so turn the whole place into a bear garden. Recently the Hull Guardians, after a long discussion, decided not to re-admit three such men to the workhouse, though they were proved to be hungry and destitute. However just it may have been, we doubt the wisdom or propriety of this decision. A workhouse should be sufficiently staffed to enable order and discipline to be enforced. The Poor-Law authorities in each large town should possess the means of isolating loafers of the Hull type who have missed their way at the commencement of their working career, and of placing them where they can be made to behave themselves, compelled to do a useful day's work for their keep, and if retained to be governed by conditions which will provide for their maintenance without any great cost to the ratepayers.

### ARTISAN CONTRIBUTORS IN AND OUTSIDE LONDON.

The claim which some supporters of the Hospital Saturday Fund have made, like Mr. Lewcock, that each subscriber to the Hospital Saturday Fund is entitled to a *quid pro quo* benefit in medical relief from any hospital receiving a grant from the Hospital Saturday Fund, rests upon no facts which will justify it. In London the workpeople's contributions, including those of the Hospital Saturday Fund, so far as the larger hospitals are concerned, amount to something less than 2 per cent. of the present ordinary income of these institutions. In the case of special hospitals they amount to something less than 3½ per cent. of the ordinary income of these institutions. It will thus be seen that so far as London is concerned the workpeople's contributions are relatively so small that the artisan class would put themselves in a very false position if they were persistently to claim any *quid pro quo*, seeing that they already receive from the Metropolitan voluntary hospitals in value enormously greater benefits, as represented by the actual cost of their treatment, than the *quid pro quo* claim would result in if it were to be admitted and enforced. We are anxious that the Hospital Saturday Fund shall continue and increase, and that is why we venture to utter this word of caution and to enforce it by a statement of the actual facts. The case of provincial hospitals is, fortunately, wholly different from that of London, for in the provinces the workpeople's collections to a particular hospital sometimes exceed 50 per cent. of its total revenue. In the provinces, too, the working classes are better organised, and have a much more accurate apprehension of the value and importance of the work done for them by the voluntary hospital. Hence the actual money given by the artisans throughout the country to these institutions will, in all probability, tend to increase, and not to diminish, each year.

### PROPOSED ISOLATION HOSPITAL FOR TYNEMOUTH.

A curious situation was disclosed lately at Tynemouth during the hearing of a public inquiry, ordered by the Local Government Board, concerning the application by the Tynemouth Corporation for power to build an isolation hospital and to acquire a large farm as part of the scheme. The Town Clerk stated in evidence that for years the Corporation has been seeking a suitable site, but every owner with land to sell refused to part with it for the purpose of having an infectious diseases hospital built there. Finally, a copyhold farm of about 130 acres, wholly situate within the borough, came into the market; and as the owner refused to sell a portion of it, the Corporation has been driven to seek permission to buy the whole. On a portion of this extensive site it is proposed to build a fever hospital, and to convert the existing Moor Park Hospital into a smallpox hospital. It is also proposed to make provision for observation cases and for advanced cases of phthisis unsuitable for sanatorium benefit under the National Insurance Act. The rest of the land—125 acres—will continue to be farmed for the present; but it is hoped eventually to reserve a portion for municipal purposes, and to convert the rest into allotments and small holdings, except a plot which it is thought could be sold on advantageous terms. It was also stated, in support of the proposal as to a smallpox hospital, that the Local Government Board itself has urged the Corporation to secure one; and that a short time ago a single case cost the Corporation £297, owing to the lack of accommodation. The Board's inspectors expressed dissatisfaction at the indefinite nature of the suggested enterprise, which they criticised as practically a speculation in land. The inspectors afterwards visited the farm and the Moor Park Hospital; but the decision of the Board has not yet been made public.

### NEW DEPARTMENT AT THE LONDON HOSPITAL.

When a few years ago it was made known that Dr. James Mackenzie was leaving Burnley for a wider professional horizon in London, the authorities at the London Hospital very wisely decided to secure his services on their staff. We should imagine that this is the only example in quite recent years of a country general practitioner being invited direct on to the staff of one of the great London teaching hospitals; though before the present era of specialisation began such a step was less exceptional. In this particular case Dr. Mackenzie had already made himself a scientific reputation for originality and research, which extended, not merely beyond Burnley, but also beyond these shores altogether. Such an achievement for a man with a large general practice in an industrial town marked him as one of those shining lights who are almost bound to gravitate sooner or later to the Metropolis. It was mainly in connection with the diseases of the heart that Dr. Mackenzie's name was made, though he had in fact also done excellent work along quite other lines. At the London Hospital he was allotted twelve beds for heart cases; and so successful has this special department proved that it has now been arranged to add to it an out-patient department, a laboratory, and two clinical assistants. Although no announcement has yet been made, we may suppose that very shortly a special assistant physician will be required for this development; a precedent for such a step exists at University College Hospital, though the management of "The London" are quite equal to creating their own precedents if they want them. The experiment of this new department will be watched with interest.

### THE ROYAL INFIRMARY, SHEFFIELD

Much regret is felt among the governors of this hospital at the resignation of Mr. John Marshall, who has been a member of the committee for thirty-seven years, during twenty-two of which he has filled the important post of chairman. In The Hospital of April 27, 1912, page 103, we published an appreciation of Mr. Marshall by a colleague. During his association with the Royal Infirmary Mr. Marshall has shown the keenest interest in every worker connected with the establishment, and we have had striking testimony of the deep sense of gratitude and affection towards the late chairman which animates every member of the past and present staff. Mr. Marshall has brought the Royal Infirmary to a high state of efficiency structurally and administratively, and it is pleasant to be able to record that probably no hospital in the land has had a chairman whose example and counsel have proved more helpful than his to successive generations of the younger men. It is fitting that his colleagues on the board should have commissioned an eminent artist to paint Mr. Marshall's portrait to hang in the board room, where there are already others of supreme interest to hospital people. In his retirement Mr. Marshall may be cheered by the knowledge that his withdrawal from active work will be felt by his fellow-workers for many years to come. It is not easy to find anyone competent and willing to accept the chairmanship of a hospital committee vacated by so great a personality. The Royal Infirmary committee have, however, been fortunate enough to secure the services of Mr. Henry H. Bedford. Mr. Bedford has always manifested the keenest interest in the work and welfare of the Royal Infirmary. His father, the late Mr. John Bedford, was a member of the infirmary board for over forty-six years, a fact which in no small measure influenced him in accepting the onerous position of chairman of the most important hospital in Sheffield. The Royal Infirmary now contains 330 beds and has twenty additional beds at its semi-convalescent home or annexe.

### THE HOME AND HOSPITAL FOR SICK CHILDREN, SYDENHAM.

Last year we dealt at some length with the work, prospects, and financial position of this children's hospital at Sydenham; and advocated an attempt to obtain more local support and interest, and abstention from too ambitious a programme. From the proceedings at the annual meeting, held on February 11, it would seem that at any rate the first part of this advice has been followed out; and not any too soon, in view of the results of the year's working, as shown by the accounts. The Mayor of Lewisham took the chair; a London County Councillor is treasurer; and there is other evidence that the authorities have realised the gravity of the position and are seeking to broaden the basis of financial support. Briefly, the expenses for the year exceeded the receipts by £462; and to meet a part of the deficiency £500 of Consols had to be sold, the first time in the forty odd years of the hospital's existence that invested funds have had to be trenched upon. We fear that in the present state of the market these Consols must have been sold at a heavy loss compared with the price originally given for them. It is pleasing to note that a determined effort is to be made to prevent this institution from going under, and we can hardly doubt that if a larger proportion of the residents in the district are made acquainted with its work and its needs, the increased support needed will soon be forthcoming. One of the criticisms we felt constrained to offer a year ago was upon the faulty arrangements for dealing with fire; and we are pleased to see that the matter has been taken in hand. It is never pleasant to criticise; but when such remarks are accepted in a sensible spirit and reforms are instituted, as in this case, the task is made much less irksome. We trust the management will find their difficulties speedily becoming less troublesome.

### "MOUNT VERNON" AND THE FUTURE.

The future of the Mount Vernon Hospital for Diseases of the Chest has been the subject of anxious deliberation for the past year and more to those who are chiefly concerned in its management. As is well known, the Society carries on an out-patient department in Fitzroy Square, a hospital at Hampstead, and a sanatorium for tuberculous cases at Northwood. Faced with a certainty of diminished revenue owing to the Insurance Act, and with a diminution in the number of probable patients too, the committee of management suggested the sale of the Hampstead property and the investment of the proceeds to endow the Northwood Sanatorium. To this the medical staff objected, and in a memorandum expressed the view that the Northwood Sanatorium should be abandoned rather than the out-patient department or the Hampstead institution. The reasons which they advanced were cogent enough, from the point of view of the staff and the profession of medicine; but failed to appreciate fully the other view-point of the trustees of a charity. Some of the suggestions of the medical staff were, however, adopted; but the original proposal to sell the Hampstead property and concentrate on Northwood is still the policy of the committee, and the governors will be asked to sanction it at the forthcoming meeting on March 12.

### THE ROYAL EDINBURGH ASYLUM, MORNINGSIDE.

This institution, so familiar to Edinburgh students and graduates, has reached the hundredth anniversary of its foundation; and the annual report by the Physician-Superintendent is also the hundredth of the series. Dr. G. M. Robertson in this report presents the main facts of the year's working with admirable precision, outspokenness, and with a certain lurking humour, which adds interest to the subject. We note that alcoholism is decreasing, and general paralysis increasing, as causes of insanity among the *clientèle* of Morningside; and the latter actually accounts for more admissions (among men) than the former in the last five years. As Dr. Robertson remarks, general paralysis is also far and away more deadly than alcoholic insanity; and is, moreover, much more preventable. Artificial feeding is, as all asylum officers know, absolutely essential for a considerable percentage of lunatics, unless the death-rate is to be allowed greatly to increase. Hitherto it has excited hardly any opposition from mental patients, and has practically never been followed by any but good effects. It would seem, however, that the suffragette campaign has led friends of patients in some cases to instigate them to oppose artificial feeding. One patient even started a hunger strike in the approved manner. As he was in good health he was allowed to starve for two days, and then the attendant was instructed to turn his back on the patient for a short time. Another patient was also persuaded to pass the "striker" a piece of bread in a surreptitious way under his bedclothes. This went on for several days, until it dawned on the hunger striker that the nurses knew more about his strike than he imagined, when he resumed his ordinary method of feeding. Dr. Robertson has satisfied himself that the whole of the ill-effects upon the health of prison strikers are due, not to artificial feeding as such, but to the violent resistance offered to it; a conclusion with which everyone who has had experience of asylum work will agree.

### THE KING AND CHICHESTER INFIRMARY.

It was announced at the annual meeting of the Chichester Infirmary Governors, on February 13, that his Majesty the King has promised to open the reconstructed building in person on August 2. Following upon this statement, the Chairman (Major Chancy) moved to empower the "trustees to realise and pay into the general account such sum as shall free that account from debt and provide a working balance." The object of this resolution, which was carried unanimously, is to enable the Board to apply through the Home Secretary for permission to add the prefix "Royal" to the name of the institution. On a previous occasion, during the lifetime of King Edward, when a similar application was made it was refused, because the institution was in debt. We have every sympathy with the Chichester Infirmary in an endeavour to merit the prefix "Royal." But we must say we do not quite see how the sale of investments, in order to free the general account of a deficit, can be regarded as a praiseworthy act. We note also that the year's working resulted in a deficit of £391. As a matter of fact, the trustees have in hand awaiting investment a sum which falls short of the debt by only £360, and it is proposed to utilise this in paying the debt. We hope that the small remaining sum will be raised without the necessity for realising investments.

### CLARE HALL SANATORIUM.

The Middlesex Districts Joint Smallpox Hospital Board are considering the question of erecting new buildings, at an estimated cost of over £6,000, to provide for the treatment of smallpox patients at their Clare Hall Hospital, Barnet. This hospital was originally used for smallpox patients only, but in view of the heavy expenses of the stand-by staff and the few cases treated, was, with the permission of the Local Government Board, altered and opened on July 15 last for the treatment of incipient cases of consumption from the districts of the constituent Councils of the Board. At present there are about 100 cases under treatment, ninety being from the Middlesex County Insurance Committee. The proposed new buildings are not to be erected at once. The Joint Board have decided to proceed only with the preparation of the plans, pending the adoption of a permanent scheme for dealing with insured persons suffering from tuberculosis; the hospital is at present only used by the Middlesex County Insurance Committee under the half-yearly sanctions of the National Insurance Commissioners. The Joint Board have made arrangements with the Metropolitan Asylums Board for the reception of any smallpox cases that may occur pending the construction of the new wards.

### SOME RECENT WINDFALLS FOR VOLUNTARY HOSPITALS.

An anonymous donor has given £1,000 to the funds of the Worthing Hospital, which will practically clear off the debt on the out-patient department, due to the recent installation of a full and complete equipment for electrical and $x$-ray work. The Cancer Hospital, Brompton, will receive £4,000 under the will of the late Mrs. Sarah Annie Ward, of York. This hospital has been exceptionally fortunate in legacies during the last year or two, and has shown itself worthy by ambitious schemes of extension for research and for treatment. The Taunton Hospital has also been fortunate; for during last year the committee not only invested £13,276, bequeathed by the late Mr. James Cook, but also a surplus on the year's work of about £1,000. This latter excess of income over expenditure is, however, accounted for very largely by two gifts of £1,000 each from the Hon. Mrs. Portman and the Hon. H. P. Portman, in memory of the late President of the Hospital, the Hon. E. W. B. Portman.

### TWO EAST END SPECIAL HOSPITALS.

The report on the year's working of St. Mary's Hospital for Women and Children, Plaistow, shows that considerable progress has been made. An efficient $x$-ray apparatus, the gift of Mr. and Mrs. H. O. Holford, has been installed and a photographic room provided. The number of beds available has increased to thirty-nine, and it has been decided to extend the usefulness of the institution by opening another ward, capable of accommodating twenty-two beds. Already some response to the necessary upkeep of these has been received, and the committee ask the public for an annual increase of £1,000 in the income. The appeal for the Nurses' Home (the Matron's Million Pennies Fund) has reached nearly half a million pennies, the total now standing at £1,874. In this connection an appeal at the Latin British Exhibition was promoted by the ladies of the hospital and Miss Ray (the matron), and was responsible for £439 11s. being added to the Fund. Lord Hollenden is the new President of the institution, in succession to the late Lord Lister. The East London Hospital for Children, better known as "Shadwell," is another East End hospital whose means are straitened by the lack of any well-to-do inhabitants in its district. From Lord Erroll's speech at the annual court of governors on February 24 it appears that only a special donation of £4,000 from one generous friend (Mr. C. F. Denny) saved the hospital from a serious deficit on the year's work. The only economy which it was possible to effect last year was the closing of the convalescent home at Bognor, a step which was stated to have made the governors exceedingly unpopular. This, too, is a hospital in very real need of more support, and one which does a great work among the children of the poor.

### GLASGOW HOSPITALS' GOOD FORTUNE.

Under the will of Miss Margaret Dolores Dunlop several Glasgow institutions receive handsome legacies. The Western Infirmary and the Royal Hospital for Sick Children get £2,000 apiece; the Broadstone Jubilee Hospital, £1,200; the District Nursing Association, Port Glasgow, £500; the Higginbotham Sick Poor Nursing Association, £500; and the Lockerbie District Nursing Association, £300. Sir William Arrol has left legacies to public institutions in Glasgow and the West of Scotland amounting to about £11,000. The two largest bequests are of £2,500 each to the Royal and the Western Infirmaries of Glasgow.

### HOSPITAL SUNDAY IN SUNDERLAND.

Considerable gratification has been aroused in hospital circles in the North by the action of the newly appointed Dean of Durham in making his first public appearance in the diocese at a special meeting in the Victoria Hall, Sunderland, in aid of the Royal Infirmary and its adjunct, the Children's Hospital. In his London pulpit Canon Hensley Henson was, of course, an eloquent pleader of the Hospital Sunday cause of the Metropolis; and it is, therefore, not surprising that his appeal at Sunderland was urged with a knowledge, understanding, and sympathy which must have proved the best possible form of introduction to the "commercial capital" of county Durham, as the Mayor claimed Sunderland to be. The hall was well filled in every part, according to the report in the local press; and we trust that this was due to a genuine interest in the local hospitals as well as to the natural desire of the Wearsiders to see how their new dean would "fettle." That this was the case would seem to be established by the Mayor's definition of Sunderland as a town celebrated for its hospitals, which are largely supported by the classes standing most in need of them. At any rate, the Dean fettled very successfully; and in the course of his remarks took occasion to deplore the party spirit which is the curse of the country just now. Indeed, Dr. Henson said he half thought of announcing a prize, to be given in ten years' time to anyone who could guess what his politics are, judging solely from his public utterances. A satisfactory collection, we note, was taken up.

### IRISH HOSPITALS AND LEGACY DUTY.

According to Mr. R. W. E. Parker, all kinds of charities in Ireland escape the payment of legacy duty under an Act about seventy years old, provided that the money is spent in Ireland. This being the case, it adds renewed weight to the claim of British hospitals to similar treatment. That claim, as we urged last week, is based first and foremost upon simple justice, long overdue; secondly, upon common gratitude to the voluntary hospitals for the part they are playing in making the Insurance Act workable; and, thirdly, it now appears, upon the principle of equal treatment for all the constituent parts of the kingdom. Unfortunately, so far it has not proved of any importance as a snarer of votes; and so for practical politicians is of little interest and no moment whatever. We look forward to a day when it shall be no longer possible for a just and necessary reform to be refused simply on the ground that other reforms, not so just and not so necessary, may be brought forward if it is granted. Meanwhile, we can only recommend unceasing effort to all who hold our hospitals to be rightly entitled to the concession now refused them, and to note the existence of yet "another injustice to Ireland"!

### INSTITUTIONAL HOUSEKEEPING.

In our correspondence columns this week is printed a letter from the Matrons and Schools Committee of the National Food Reform Association, in which appears a series of statements concerning institutional housekeeping and cookery which we can fully confirm and appreciate. That The Hospital has already for some time done its best to arouse interest in this topic, and to improve as far as possible the present not at all satisfactory state of affairs, is a coincidence to which we should not allude were it not that it affords such convincing evidence of our practical sympathy with the aims and ideals of the Food Reform Association. The part of the problem which, so far, we have attempted to tackle, in the section devoted to Institutional Housekeeping, is that of encouraging those who already have this work to do to improve themselves in it, to take a pride and an interest in it; and of arousing, as far as may be, a spirit of emulation and of efficiency. What degree of success has been attained is for others to measure. The Food Reform Association's Committee puts forward a programme which goes yet deeper into the heart of the matter. Their schemes are educational, and as such they have an inherent advantage which renders them full of promise. After all, to train the future institutional housekeeper from the start is the one way which offers a chance of real progress; and as such the scheme has our hearty sympathy for its establishment and its success.

### MISS FISHER, OF LEEDS.

The retirement of Miss Fisher, who has been connected for over twenty-six years with the Leeds General Infirmary, during twenty-three of which she has held the post of lady superintendent, ends a memorable work by one of the most successful of British matrons. When we inspected the Leeds General Infirmary some two years ago, we found Miss Fisher as active and energetic as ever, full of ambition to improve and extend the efficiency of the work for which she was responsible. Miss Fisher followed Miss Gordon, who was one of the best of women and wisest of superintendents, whose work at St. Thomas's Hospital is memorable, and will live. Miss Fisher fortunately inherited the spirit, wisdom, and energy of her predecessor, and it is no small triumph for a great woman worker like Miss Fisher that she should have carried on so successfully and thoroughly her life's work at Leeds, for the Leeds General Infirmary is one of the most onerous positions open to the ablest workers in the nursing world. Miss Fisher came to Leeds from the Liverpool Royal Infirmary, one of the very best of voluntary hospitals in the North of England. We are confident we do but voice the sentiments of the authorities at Liverpool and Leeds when we say that those who know most of Miss Fisher's services and work place the highest value upon them. They will always regard her as one of the foremost hospital matrons of modern times. We wish her long life and many healthful, pleasurable days.

### THE NEW MATRON AT LEEDS INFIRMARY.

There were a large number of applicants for the matronship of the Leeds General Infirmary. The selected list included Miss H. H. Crook, of the Royal Infirmary, Bradford, Miss E. I. Innes, of the Royal Infirmary, Halifax, Miss Lucy Maclean, of the Western Infirmary, Glasgow, and Miss M. P. Scovell, of the General Hospital, Swansea. The committee at their meeting on February 21 elected Miss Innes, who is at present matron of the Royal Infirmary, Halifax. Miss Innes was trained at the Leeds General Infirmary, where she held the post of sister in several departments, and for three years held the office of assistant matron. She was also for two years assistant matron at Halifax. Miss Innes ought to make an ideal successor to Miss Fisher, as every one who takes the trouble to refer to the report on the Royal Halifax Infirmary, published in The Hospital of February 12, 1910, will, we have little doubt, agree. We heartily congratulate the committee of the Leeds General Infirmary upon the choice they have made, and Miss Innes upon her selection.

### BATH AND ITS BATHS.

It is interesting to record, in view of the Note appearing in last week's issue relative to "Bath and its Bathers," that definite proposals have now been brought forward by the Development Sub-Committee for the improvement of the Bathing Establishment. It is proposed that a syndicate should be given the option of building a large hotel situate facing the Institution Gardens, and that Mr. A. J. Taylor be asked to prepare sketch plans for new Corporation Baths to be built between the present Roman Promenade and the above-mentioned proposed hotel. We trust that the result will be to place Bath in the front rank of British curative resorts in the matter of equipment; and that balneotherapy will shortly be no longer one of those things they do better abroad. It is also announced that, in the event of the failure of negotiations now proceeding with a syndicate, the City of Bath will undertake an independent scheme of development to make the most of the radio-activity lately shown to exist in the waters.

### HEALTH WEEK.

The National Health Week Committee have fixed April 6-12 as the dates for this year's "Health Week." The purpose of the campaign is, as before, to educate the public generally to the fact that disease to a large extent can, and should, be prevented, and to impress upon them the need and meaning of public and personal hygiene, and to stir up public opinion with regard to high disease rate and infantile mortality. An appeal is being made to every class of the community for help and co-operation, to local authorities, to the medical and nursing professions, to the clergy and teachers, and to every kind of friendly society and guild, that each of these to the best of their ability, whether it be by lecture or Sunday-school lesson, may help to bring home to adult and child alike that which is of vital interest to the nation—the preservation of national health.

### THE FINANCE OF SANATORIUM BENEFIT.

The London Insurance Committee is very seriously concerned about the resources placed at its disposal for the purpose of providing sanatorium benefit for the insured of the county. At its recent meeting, it was publicly stated that the funds to which the Committee is entitled for this purpose will not be sufficient for ordinary purposes (*i.e.*, for sending tuberculous insured to sanatoria when that course is desirable); much less, therefore, will they allow of the extension of sanatorium benefit to the dependants of insured persons, which is permissible under the Act when a surplus exists. In these circumstances the Committee decided to draw the attention of the Treasury at once "to the fact that the funds now available under existing conditions for defraying the cost of sanatorium benefit, other than domiciliary treatment, for insured persons resident within the County of London are inadequate." Further, the Treasury is asked to provide immediately other funds to supplement the sums originally available for all forms of treatment. The Committee's action is to be communicated to the Insurance Committees of the large provincial towns, with a view to their co-operation in the matter. We realise also that for the present dispensary and similar out-patient treatment for tuberculosis is out of the question in London, owing to the same financial difficulty. We sympathise with the despair of the London Committee over its task of making bricks without straw.

### A RETURNED RED CROSS UNIT.

On Saturday last the Women's Sick and Wounded Convoy Corps attended a Territorial field-day at Radlett and took charge of the dressing station, where they treated a number of casualties from both forces. Among the members who were present were many of those who took part in the Corps expedition to Kirk Kilisse, and who were, therefore, fully acquainted with war service. Mrs. Godfrey, who superintended the camp ovens, was the centre of attraction; she had covered herself with glory by her expert handling of the commissariat department at the front, where she, single-handed, provided meals for the Corps base hospital with nearly a hundred patients. To those who witnessed Saturday's display it must be evident that such an organisation should do its utmost to develop to the full that side of its activities which is now represented by Mrs. Godfrey and her two assistant cooks. Camp cookery is a much misunderstood art, and no voluntary aid detachment should think it beneath its dignity to master the rudiments of that art so as to be able, when occasion arises, to deal as effectively with commissariat emergencies as the Corps dealt with them in Bulgaria. In our opinion the Women's Sick and Wounded Convoy Corps would be well advised to lay special stress on the importance of the thorough study of camp cookery by all its members. First aid and the essentials of nursing are easily learned, and recruits can rapidly be drilled so as to become effective stretcher-bearers; but it is far less easy to train members to be good and economical camp cooks. What the Corps accomplished in the Bulgarian War is graphically told by its Commandant, Mrs. St. Clair Stobart, in the current number of the *Contemporary Review*. It is interesting to note that Mrs. Stobart, since her return from the front, has accepted a Progressive invitation to stand as a candidate for the Westminster division at the forthcoming London County Council election.

### RETIREMENT OF A VETERAN

The Wandsworth Board of Guardians has decided to grant Dr. J. Breward Neal, medical superintendent of St. John's Hill Infirmary, an annual retiring allowance of £404 7s. 7d. Dr. Neal has been in the Poor-Law service for thirty-four years, the greater part of which time he has spent at Wandsworth, and the Guardians have recognised his work by adding six years to his actual service in calculating his pension. Dr. Neal has had the supervision of one of the old-style infirmaries, often greatly overcrowded, and involving considerable skill in the management. His duties have been lightened during the past two years since the opening of the new St. James' Infirmary at Wandsworth, although the number of patients under his charge has been over 600. The Board has, further, paid a high compliment to Dr. Neal by recording on the minutes their appreciation of and thanks for his services.

